# A C-Type Lectin Highly Expressed in *Portunus trituberculatus* Intestine Functions in AMP Regulation and Prophenoloxidase Activation

**DOI:** 10.3390/antibiotics10050541

**Published:** 2021-05-07

**Authors:** Yuan Liu, Yue Su, Ao Zhang, Zhaoxia Cui

**Affiliations:** 1 CAS and Shandong Province Key Laboratory of Experimental Marine Biology, Institute of Oceanology, Chinese Academy of Sciences, Qingdao 266071, China; suyuewan199532@163.com (Y.S.); aozaizz@163.com (A.Z.); 2 Laboratory for Marine Biology and Biotechnology, Qingdao National Laboratory for Marine Science and Technology, Qingdao 266071, China; 3 Center for Ocean Mega-Science, Chinese Academy of Sciences, Qingdao 266071, China; 4 University of Chinese Academy of Sciences, Beijing 100049, China; 5 School of Marine Science, Ningbo University, Ningbo 315211, China; cuizhaoxia@nbu.edu.cn

**Keywords:** C-type lectin, *Portunus trituberculatus*, innate immune recognition, AMP expression, proPO system

## Abstract

A C-type lectin (PtCLec2) from *Portunus trituberculatus* was identified for characterization of its role in defense and innate immunity. PtCLec2 contains a single carbohydrate-recognition domain (CRD) with a conserved QPD motif, which was predicted to have galactose specificity. The mRNA expression of PtCLec2 was predominantly detected in intestine and increased rapidly and significantly upon pathogen challenge. The recombinant PtCLec2 (rPtCLec2) could bind various microorganisms and PAMPs with weak binding ability to yeast and PGN. It agglutinated the tested Gram-negative bacteria (*Vibrio alginolyticus* and *Pseudomonas aeruginosa*), Gram-positive bacteria (*Staphylococcus aureus* and *Micrococcus luteus*), and rabbit erythrocytes in the presence of exogenous Ca^2+^, and these agglutination activities were suppressed by LPS, d-galactose, and d-mannose. Further, rPtCLec2 enhanced phagocytosis and clearance of *V. alginolyticus*, and displayed inhibitory activities against the tested bacteria. Knockdown of PtCLec2 decreased the transcription of two phagocytosis genes (PtArp and PtMyosin), three prophenoloxidase (proPO) system-related genes (PtPPAF, PtcSP1, and PtproPO), six antimicrobial peptides (AMPs) (PtALF4-7, PtCrustin1, and PtCrustin3), and PtRelish but upregulated the expression levels of PtJNK, PtPelle, and PtTLR. These results collectively indicate that PtCLec2 might perform its immune recognition function via binding and agglutination, and mediate pathogen elimination via regulating hemocyte phagocytosis, AMP synthesis, and proPO activation.

## 1. Introduction

C-type lectins, a large group of Ca^2+^-dependent carbohydrate-binding proteins, function as pattern recognition receptors (PRRs) to identify specific carbohydrates on the surface of heterologous microorganisms (pathogen-associated molecular patterns, PAMPs) [1,2]. C-type lectins typically contain at least a carbohydrate-recognition domain (CRD) of approximately 110–130 amino acids. The CRD is characterized by a double-loop structure formed by two conserved disulfide bridges [3]. Four Ca^2+^-binding sites are present in the CRD, and only site 2 is considered to participate in carbohydrate binding.

In vertebrates, C-type lectins could be divided into two main groups based on the conserved motifs in Ca^2+^-binding site 2. The C-type lectins containing QPD (Gln–Pro–Asp) motif are specific for galactose binding, while the lectins with EPN (Glu–Pro–Asn) motif are mannose-specific C-type lectins [3]. However, the carbohydrate specificity of these conserved motifs might be more diverse in invertebrates [4]. For example, PmLec with QPD motif from *Penaeus monodon* could have binding affinity to galactose and glucose [5], and LvCTL1 with EPN motif from *Litopenaeus vannamei* could bind and recognize mannose, galactose, and glucose [6]. Furthermore, several variations of motifs have been found in invertebrates, such as QPG (Gln–Pro–Gly), QPN (Gln–Pro–Asn), QPS (Gln–Pro–Ser), QPT (Gln–Pro–Thr), YPT (Thr–Pro–Thr), EPD (Glu–Pro–Asp), EPS (Glu–Pro–Ser), EPK (Glu–Pro–Lys), and EPQ (Glu–Pro–Gln) [7,8].

C-type lectins could interact specifically with carbohydrates on the surface of microorganisms and activate host defense mechanisms to effectively resist the invasion of pathogenic microorganisms [9]. Recently, a number of C-type lectins, expressed mainly in hepatopancreas and hemocytes, have been reported in crustaceans [10]. They have been observed not only to function in pathogen recognition, microbial combination, and agglutination [11] but also to induce subsequent innate immune responses, such as opsonization, activation of prophenoloxidase (proPO) system, and regulation of antimicrobial peptides (AMPs). For instance, PcLec3 from *Procambarus clarkii* and FmLdlr from *Fenneropenaeus merguiensis* promoted phagocytosis of hemocytes and clearance of *Vibrio* [12,13]. LvCTL5 from *L*. *vannamei* and MjCC-CL from *Marsupenaeus japonicus* could participate in antibacterial immunity response by regulating the expression of AMPs [14,15]. EsLecB from *Eriocheir sinensis* and PcLec2 from *P. clarkii* could perform as an upstream factor of the proPO system [16,17]. Compared with other crustaceans [18,19,20,21], little is known about the mechanisms involved in regulation of AMP expression or proPO activation by C-type lectins in *Portunus trituberculatus*.

In the present study, a novel C-type lectin (PtCLec2) from *P. trituberculatus*, highly expressed in the intestine, was cloned and characterized. The main objectives of this study were to identify its domain structure; detect its mRNA expression patterns in tissues and in response to pathogen challenge; investigate the immune function of recombinant PtCLec2 (rPtCLec2), including in PAMP and microorganism binding, bacterial agglutination, hemagglutination, antimicrobial activity, bacterial clearance activities, and phagocytosis; and explore its potential role in the activation of proPO and regulation of AMP expression and signal pathway by RNA interference (RNAi).

## 2. Results

### 2.1. Cloning and Characterization of PtCLec2 cDNA

**Figure 1:**
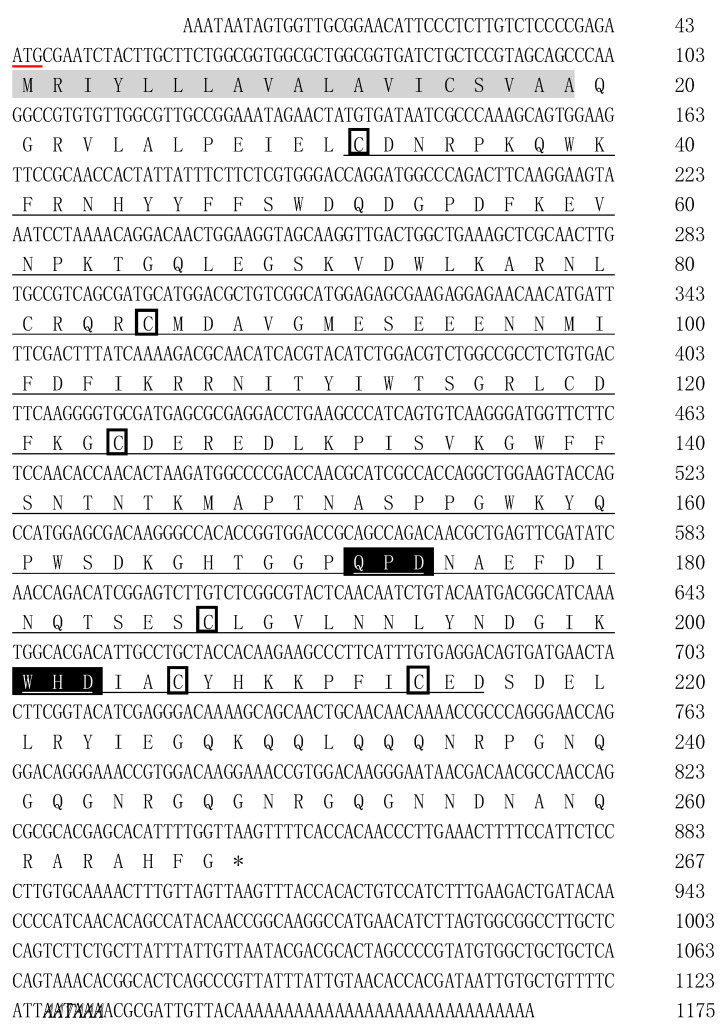
Nucleotide sequence and deduced amino acid sequence of PtCLec2. The start and stop codons are indicated by red line and asterisk, respectively. The predicted signal peptide is shown in gray shading and the CRD region is underlined in black. The motifs (QPD and WHD) of Ca^2+^-binding site 2 are shown with black shading. Positions of conserved cysteines in the CRD are indicated by boxes. The poly(A) tail signal sequence (AATAAA) is marked with bold-italic letters.

The full-length cDNA sequence of PtCLec2 was cloned by RACE-PCR and submitted to GenBank under accession no. MN394840. It was 1175 bp in length and consisted of a 5′-untranslated region (UTR) of 43 bp, a 3′-UTR of 328 bp, and an open reading frame (ORF) of 804 bp. A canonical polyadenylation signal AATAAA was found 13 nucleotides upstream of a poly(A) tail. The ORF of PtCLec2 encoded a polypeptide of 267 amino acid residues including a typical N-terminal signal peptide (residues 1–19) and a single CRD with QPD (Gln^172^–Pro^173^–Asp^174^) and WHD (Trp^201^–His^202^–Asp^203^) motifs. Six cysteine residues were found in the CRD, including four conserved cysteine residues (Cys^124^, Cys^187^, Cys^206^, and Cys^214^) forming two internal disulfide bridges and two additional cysteine residues (Cys^32^ and Cys^85^) located at the N-terminus. The mature PtClec2 was estimated to be 30.65 kDa and the theoretical isoelectric point was 7.53 (Figure 1).

### 2.2. Multiple Alignment and Phylogenetic Analysis of PtCLec2

**Figure 2:**
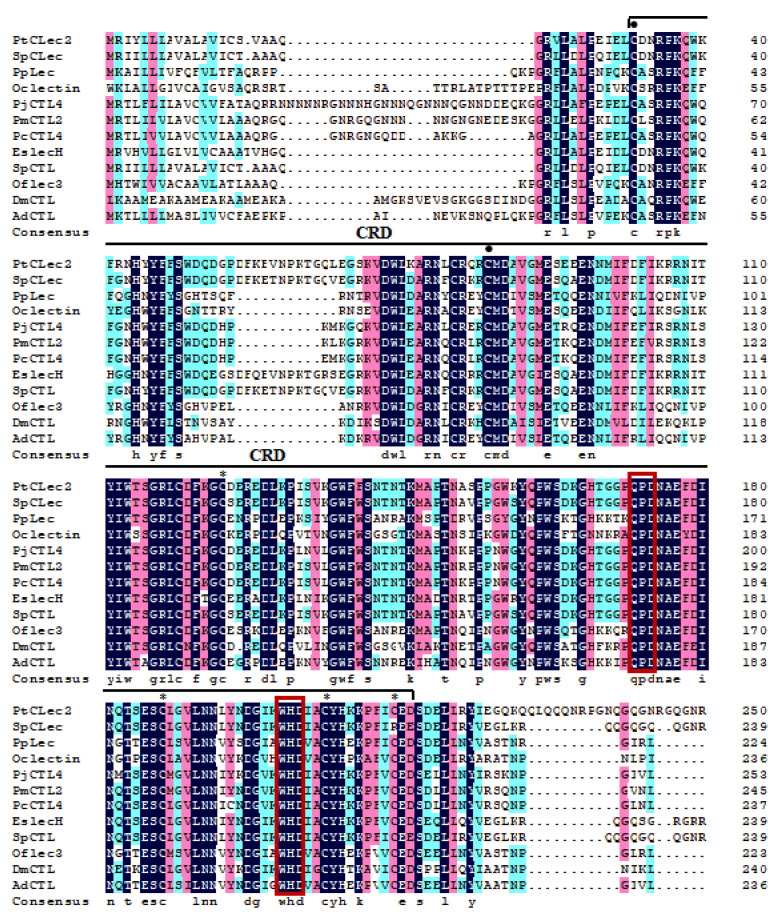
Multiple alignment of PtCLec2 with other arthropod C-type lectins. The CRD domain enclosed with bracket. The conserved amino acids are shaded in black, and the similar residues are indicated in blue or red. The four conserved cysteines are labeled with asterisks (*), and two additional cysteines are labeled with dark spots (•). The conserved Ca^2+^-binding site motifs (QPD and WHD) are marked by red boxes. The sequences include SpCLec (*S. paramamosain*, AGC96521.1), PpLec (*P. plagipennis*, ATU82908.1), Oclectin (*O. cincta*, ODM99338.1), PjCTL4 (*P. japonicus*, AHA85979.1), PmCTL2 (*P. merguiensis*, AGS42193.1), PcCTL4 (*P. chinensis*, ACJ06432.1), EsLecH (*E. sinensis*, APA32005.1), SpCTL (*S. paramamosain*, SAY41335.1), Oflec3 (*O. furnacalis*, AIR96004.1), DmCTL (*D. magna*, KZS16226.1), and AdCTL (*A. darlingi*, ETN63566.1).

**Figure 3:**
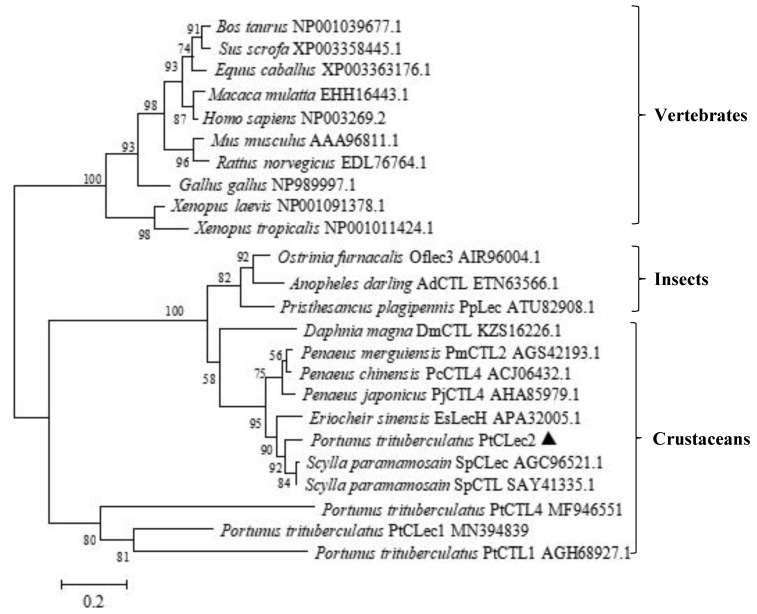
Neighbor-joining phylogenetic analysis based on amino acid sequences of CRDs from C-type lectins. Numbers in each branch indicated bootstrap values for 1000 replicates. PtCLec2 is marked with a triangle (▲).

BLAST analysis indicated obvious similarity between PtCLec2 and other members of C-type lectins (Figure 2), such as SpCTL from *Scylla paramamosain*, (SAY41335.1, 90%), SpCLec from *S*. *paramamosain* (AGC96521.1, 89%), EsLecH from *E. sinensis* (APA32005.1, 82%), PcCTL4 from *Penaeus chinensis* (ACJ06432.1, 77%), PjCTL4 from *P*. *japonicus* (AHA85979.1, 75%), and PmCTL2 from *P. merguiensis* (AGS42193.1, 75%). Multiple alignment of 12 arthropod C-type lectins revealed they all had a single CRD domain containing four traditional cysteines and conserved QPD and WHD motifs. Two additional cysteines were found at the N-terminus of CRDs, suggesting these CRDs belong to the long form. The phylogenetic tree topology showed that the analyzed C-type lectins fell into two clades (Figure 3). One clade was the invertebrate clade including C-type lectins from insects and crustaceans, while another vertebrate clade consisted of C-type lectins from amphibians, birds, and mammals. PtCLec2 was not grouped with other C-type lectins from *P. trituberculatus*. PtCLec2 was firstly clustered with C-type lectins from *Scylla paramamosain* to form a sister relationship with EsLecH from *E. sinensis*.

### 2.3. Quantitative Analysis of PtCLec2 Transcripts

**Figure 4:**
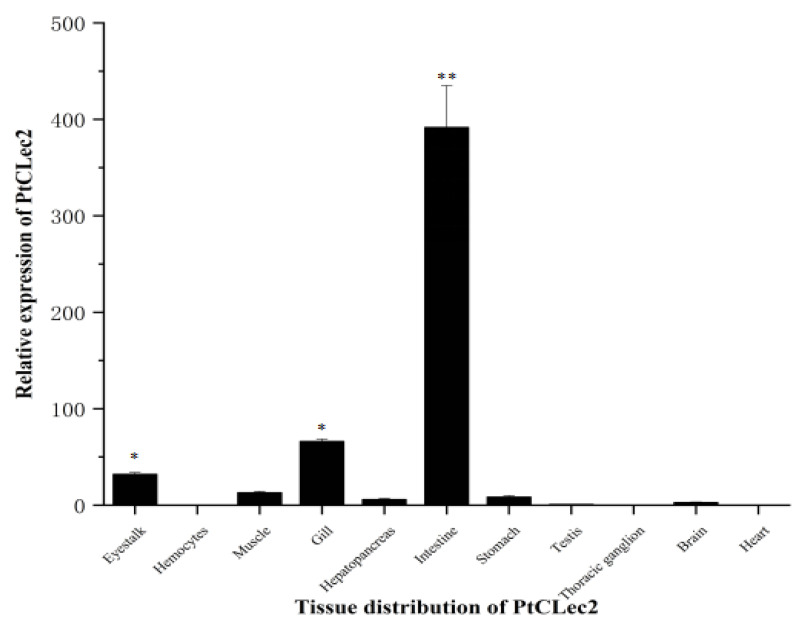
Expression levels of PtCLec2 determined by qRT-PCR in tissues of crabs. Error bars represent the mean ± S.D. (*n* = 4). The expression levels in brain, eyestalk, gill, heart, hepatopancreas, intestine, muscle, stomach, thoracic ganglia, and testis are relative to that of hemocytes (* *p* < 0.05, ** *p* < 0.01).

qRT-PCR was performed to detect the mRNA expression of PtCLec2 in 11 tissues of healthy crabs. PtCLec2 was highest expressed in intestine, which was 194.5-fold higher than that in hemocytes (*p* < 0.01). The mRNA expression levels of PtCLec2 in gill and eyestalk were also higher while relatively lower expression in muscle, stomach, hepatopancreas, brain, testis, heart, and thoracic ganglia (Figure 4).

**Figure 5:**
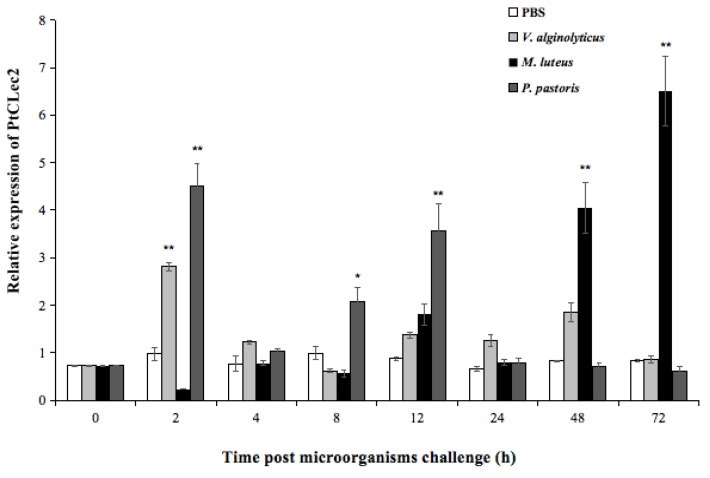
Temporal expression of PtCLec2 in hemocytes of crabs post-microorganism challenge. Error bars show the mean ± S.D. (*n* = 3). Asterisks indicate significant differences compared to the control group at the same sampling time (* *p* < 0.05, ** *p* < 0.01).

The temporal mRNA expression levels of PtCLec2 in hemocytes were significantly upregulated upon challenge with *V. alginolyticus*, *M. luteus*, and *P. pastoris* (Figure 5). After *V. alginolyticus* stimulation, the mRNA expression of PtCLec2 significantly increased to 2.2-fold of the control level at 2 h post-injection (*p* < 0.01). Then, PtCLec2 expression dropped significantly and returned to the control level from 4 to 72 h post-stimulation. PtCLec2 transcripts showed two peaks in expression profile after *P. pastoris* challenge. It first peaked at 2 h post *P. pastoris* challenge, which was 4.1-fold to that of control (*p* < 0.01), declined at 4 h post-stimulation, and gradually increased at 8 h post-stimulation (1.2-fold of control, *p* < 0.05). After that, PtCLec2 transcripts peaked again at 12 h post-stimulation (3.4-fold of control, *p* < 0.01) and recovered to the control level at 24 h post-stimulation. The expression of PtCLec2 was upregulated until 12 h post *M. luteus* challenge and increased to the highest level at 72 h post-stimulation (6.7-fold of control, *p* < 0.01).

### 2.4. Expression and Purification of rPtCLec2

**Figure 6:**
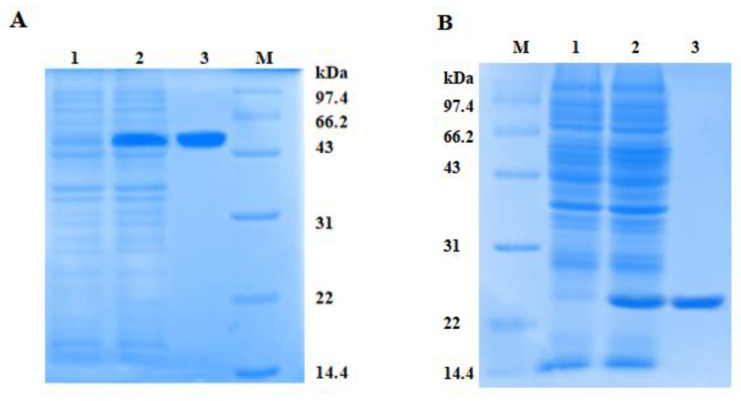
Expression and purification of rPtCLec2 (**A**) and rTrx (**B**) by SDS-PAGE analysis. Lane M, molecular marker (kDa); lane A1, negative control of rPtCLec2 without induction; lane A2, induced rPtCLec2 by IPTG; lane A3, purified rPtCLec2. Lane B1, negative control of rTrx without induction; lane B2, induced rTrx by IPTG; lane B3, purified rTrx.

To investigate the potential activities of PtCLec2, rPtCLec2 was expressed in an *E. coli* expression system as an inclusion body (Figure 6). In accordance with the predicted molecular mass, rPtCLec2 containing the extra His-tag and Trx-tag had an obvious band with molecular mass of approximately 50 kDa. rTrx was also induced and purified from pET32a vector and served as a control. The concentration of rPtCLec2 and rTrx was 1.39 and 1.12 mg mL^−1^, respectively.

### 2.5. PAMP-Binding Activity of rPtCLec2

**Figure 7:**
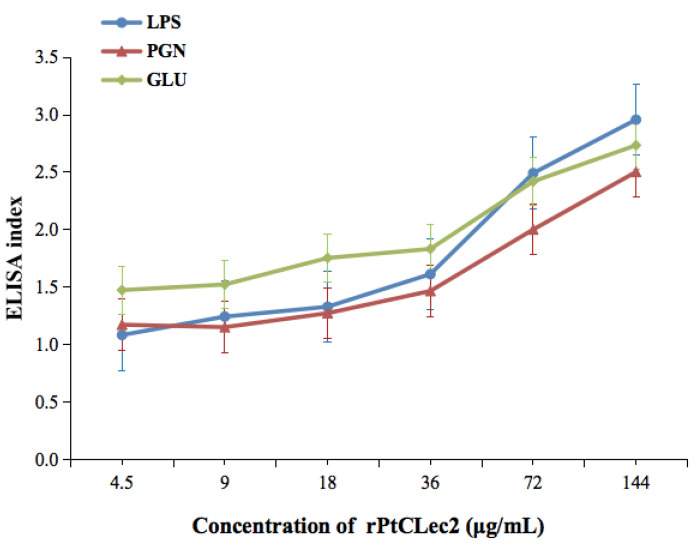
Binding activities of rPtCLec2 to PAMPs by ELISA analysis. ELISA index values above 1.0 are considered to be positive. Data shown as the mean ± S.D. derived from three independent repeats. LPS: lipopolysaccharide, PGN: peptidoglycan, GLU: glucan.

LPS, GLU, and PGN were used to test the binding of rPtCLec2 to microorganism components. As shown in Figure 7, the EI values of rPtCLec2 toward LPS, GLU, and PGN were above 1.0 at the minimum concentration of rPtClec2 (4.5 μg mL^−1^) and the binding of rPtCLec2 showed a dose-dependent trend. Compared with binding to LPS and GLU, rPtClec2 showed relatively weak affinity for PGN. The control protein rTrx had no PAMP-binding capacity (data not shown).

### 2.6. Microorganism-Binding Activity of rPtCLec2

**Figure 8:**
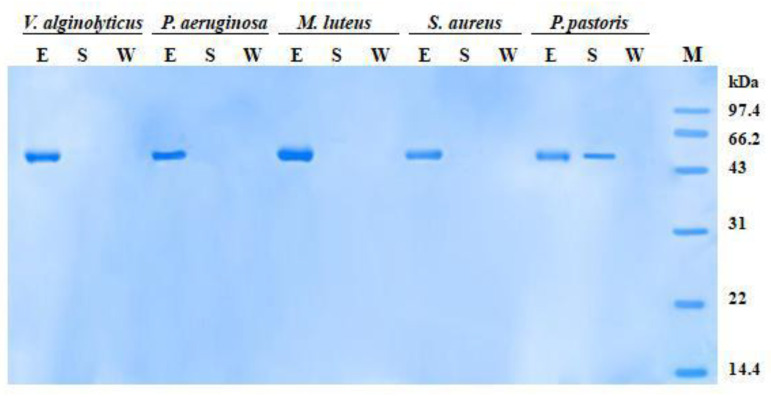
Microbial-binding activity of rPtCLec2 revealed by SDS-PAGE analysis. Formaldehyde-fixed microorganisms were incubated with the recombinant proteins. The eluted (E), supernatant (S), and wash (W) fractions were separated to detect the interaction between rPtCLec2 and microorganisms.

In microbial-binding assay, when incubated with bacteria in the presence of Ca^2+^, rPtCLec2 protein was found in the eluted fractions but not in the supernatant and wash fractions (Figure 8). This shows that rPtCLec2 could bind all four tested Gram-negative bacteria (*V. alginolyticus* and *Pseudomonas aeruginosa*) and Gram-positive bacteria (*M. luteu* and *Staphylococcus aureus*). However, clear bands were identified in both eluted and supernatant fractions of yeast *P. pastoris*, suggesting rPtCLec2 could weakly bind to yeast. The control protein rTrx had no microorganism-binding ability due to no band being observed in the eluted fraction but there was a clear band in the supernatant fraction (data not shown).

### 2.7. Agglutinating Activity of rPtCLec2 to Microorganisms and Erythrocytes

**Figure 9:**
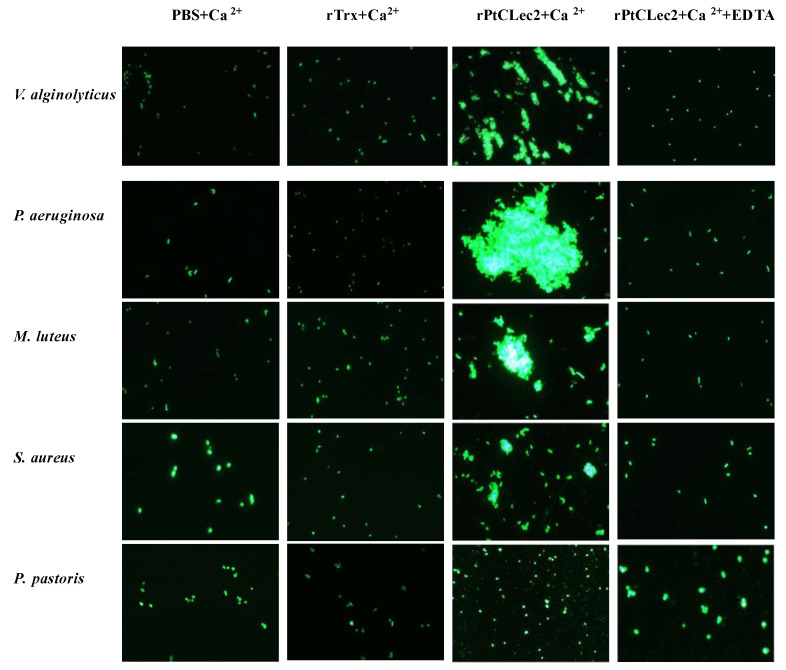
Agglutinating activity of rPtCLec2 toward microorganisms. The FITC-labeled bacteria and fungi were incubated with rPtCLec2 in the presence of 10 mmol L^−1^ CaCl_2_ or 10 mmol L^−1^ EDTA.

**Figure 10:**
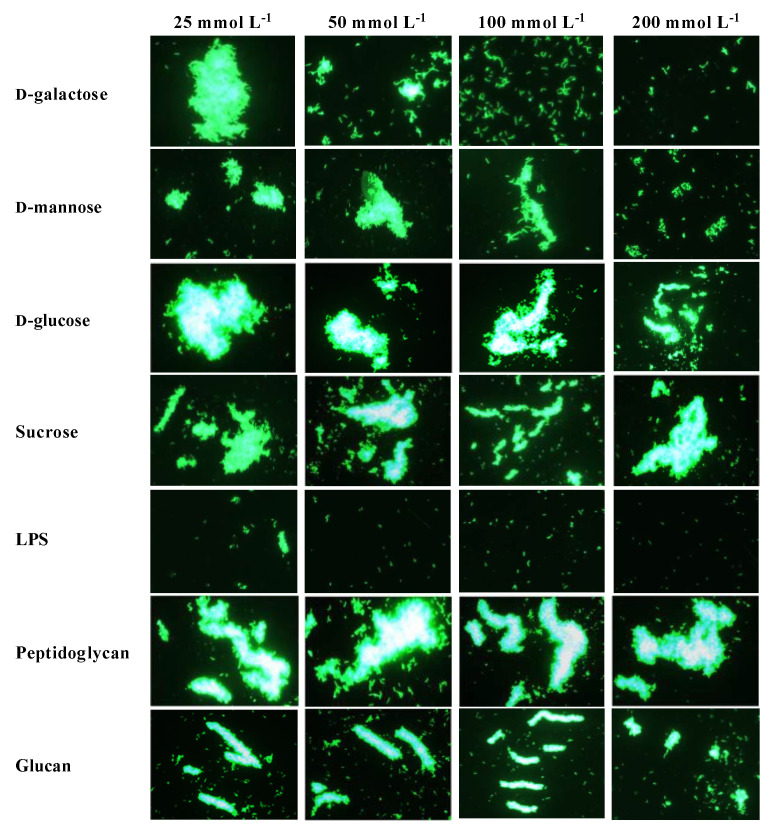
Carbohydrate-binding specificity of rPtCLec2 toward FITC-labeled *P. aeruginosa*. rPtCLec2 with different carbohydrates were incubated with FITC-labeled *P. aeruginosa* in the presence of 10 mmol L^−1^ CaCl_2_.

The microbial agglutination assay showed rPtCLec2 had obvious agglutination activity to the tested microorganisms except for fungi in the presence of Ca^2+^ (Figure 9). Moreover, rPtCLec2 had stronger agglutination ability to Gram-negative bacteria than to Gram-positive bacteria. The microbial agglutination of rPtCLec2 could completely disappear with incubation of Ca^2+^ and EDTA, indicating that this activity was Ca^2+^-dependent. To investigate carbohydrate specificity of PtCLec2, an agglutination inhibition assay was carried out (Figure 10). The agglutinating activity of rPtCLec2 against Gram-negative bacteria *P. aeruginosa* was inhibited with the increasing concentration of LPS, d-galactose, and d-mannose. However, this agglutination has no obvious change after the addition of sucrose, peptidoglycan, and glucan at the tested concentration.

**Figure 11:**
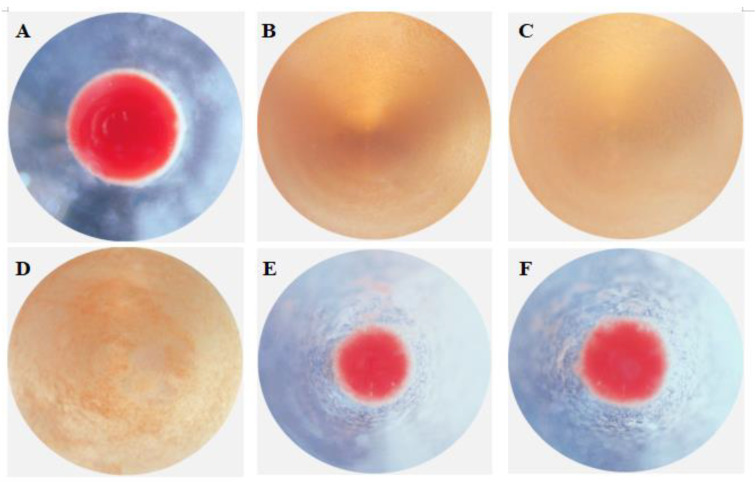
Agglutinating activity of rPtCLec2 to rabbit erythrocytes: (**A**) 1.0 mg mL^−1^ rTrx, (**B**) 1.0 mg mL^−1^ rPtCLec2, (**C**) 0.875 mg mL^−1^ rPtCLec2, (**D**) 0.75 mg mL^−1^ rPtCLec2, (**E**) 0.5 mg mL^−1^ rPtCLec2, and (**F**) 1.0 mg mL^−1^ rPtCLec2 and 10 mmol L^−1^ EDTA.

The hemagglutinating activity toward rabbit erythrocytes was shown in Figure 11. The obvious hemagglutination was found after the incubation of rPtCLec2 at the concentration of 0.75–1.0 mg mL^−1^ in the presence of Ca^2+^. However, no agglutinating effect was found when rPtCLec2 was at 0.5 mg mL^−1^ and EDTA was added.

### 2.8. Antibacterial Activity of rPtCLec2

The minimal growth inhibitory concentrations (MICs) of rPtCLec2 were determined and are summarized in Table 1. rPtClec2 was capable of inhibiting the growth of the tested Gram-negative bacteria (*V. alginolyticus* and *P. aeruginosa*) and Gram-positive bacteria (*M. luteus* and *S. aureus*). However, no significant antimicrobial activity was found toward fungus (*P. pastoris*). The highest inhibitory effect of rPtCLec2 was toward *M. luteus* with MIC values of 0.43–0.86 μM.

### 2.9. In Vivo V. alginolyticus Clearance Activity of rPtCLec2

**Figure 12:**
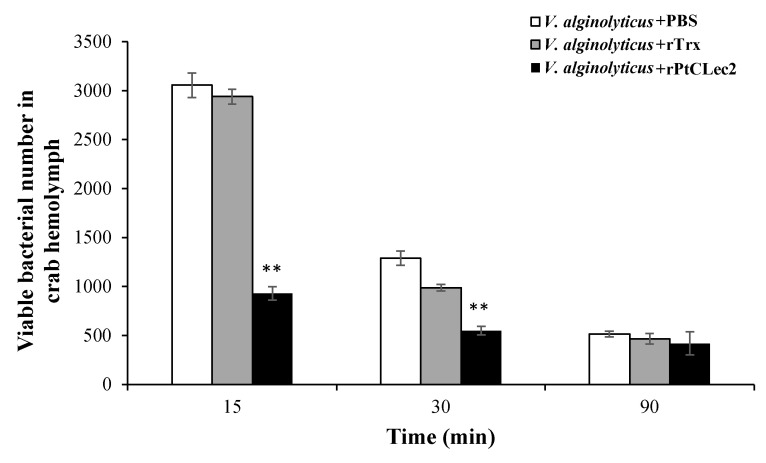
*V. alginolyticus* clearance enhanced by rPtCLec2. Crabs were challenged with rPtCLec2, rTrx, or PBS pre-coated *V. alginolyticus*. Hemolymph was collected at 15, 30, and 90 min after *V. alginolyticus* injection. The bacterial counts in 100 μL hemolymph were recorded, and the experiment was repeated three times. Significant differences compared with control are denoted with asterisks (** *p* < 0.01).

The opsonic activity of PtCLec2 was tested by the ability of hemolymph to clear *Vibrio* (Figure 12). The number of *V. alginolyticus* in hemolymph was rapidly eliminated in the rPtCLec2 group at 15 and 30 min post-injection, in which *V. alginolyticus* was pre-coated with rPtCLec2. The results reveal that PtCLec2 could increase bacterial clearance in crabs. No obvious difference was found at 90 min post-injection compared the rPtCLec2 group to the control group.

### 2.10. Phagocytic Activity of rPtCLec2

**Figure 13:**
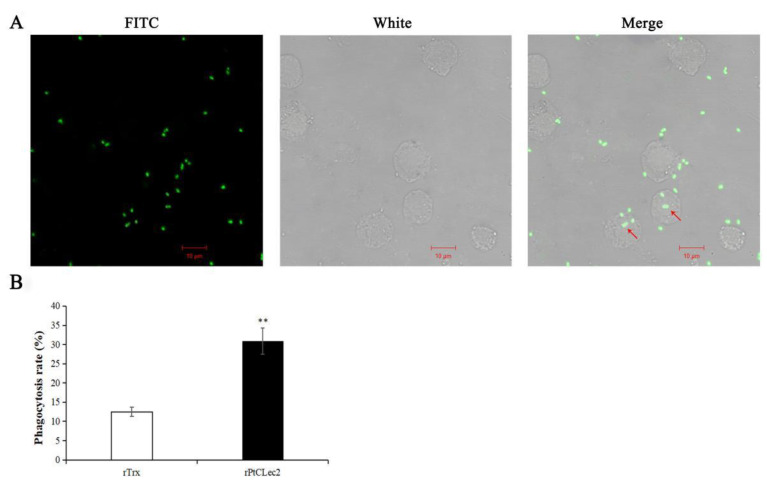
Phagocytic activity against FITC-labeled *V. alginolyticus* enhanced by rPtCLec2. A: hemocyte phagocytosis observed under fluorescence microscope; B: the phagocytic rate measured by flow cytometry. Error bars represent the mean ± S.D. of three replicates. Significant differences compared with control are denoted with two asterisks (**) at *p* < 0.01.

The hemocyte-mediated phagocytosis of *V. alginolyticus* was significantly enhanced by rPtCLec2 (Figure 13). After incubation with rPtCLec2, the phagocytized *V. alginolyticus* was detected in the cytoplasm of crab hemocytes. The phagocytic rate against *V. alginolyticus* in the rPtCLec2 group was 30.9%, which was much higher than that in the control group (12.5%).

### 2.11. Gene Knockdown of PtCLec2

**Figure 14:**
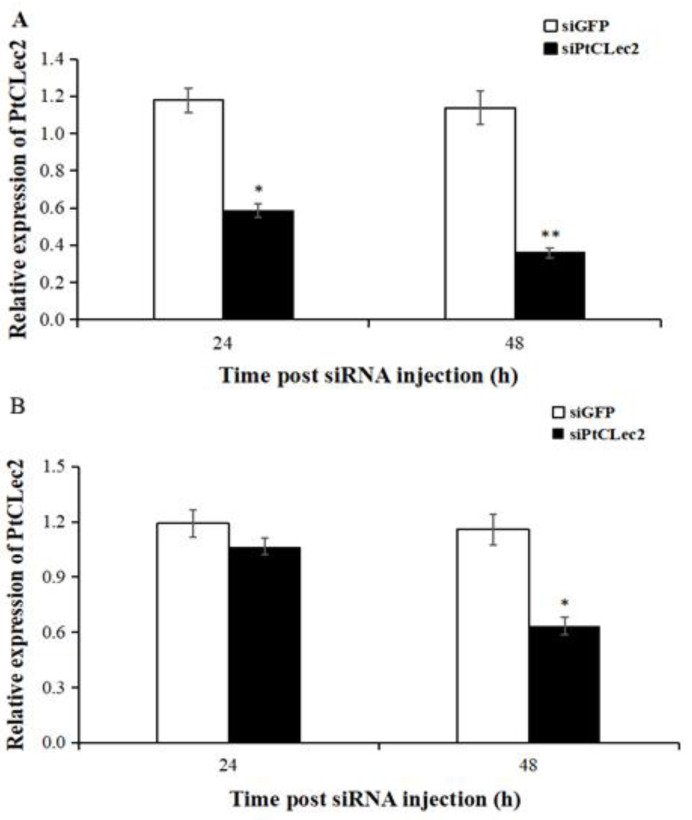
Relative expression levels of PtCLec2 in hemocytes (**A**) and intestine (**B**) at 24 and 48 h after injection with siRNA. Error bars represent the mean ± S.D. (*n* = 3). Significant differences compared with control are denoted with asterisks (* *p* < 0.05, ** *p* < 0.01).

The transcriptional levels of PtCLec2 were detected in hemocytes and intestine post PtCLec2-siRNA injection (Figure 14). The expression levels of PtCLec2 in hemocytes were significantly downregulated at 24 and 48 h post PtCLec2-siRNA injection compared to GFP-siRNA group. The higher knockdown efficiency of PtCLec2-siRNA reached up to 76.6% in hemocytes at 48 h post-injection. Hence, we used hemocytes at 48 h post siRNA injection for further interference experiments.

### 2.12. PtCLec2 Regulates the Expression of Genes Involved in Phagocytosis and Complement System

**Figure 15:**
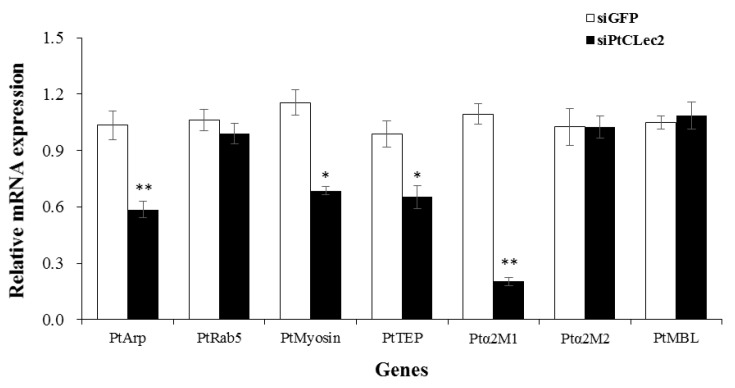
Expression levels of phagocytosis genes and complement-like genes after PtCLec2 RNAi. The control group was injected with siGFP. Error bars represent the mean ± S.D. (*n* = 3). Significant differences compared with control are denoted with asterisks (* *p* < 0.05, ** *p* < 0.01).

After knockdown of PtCLec2, the relative expression levels of phagocytosis genes PtArp and PtMyosin and complement-like genes PtTEP and Ptα2M1 were significantly decreased compared with the GFP-siRNA group (Figure 15). However, no significant effect was found on the expression levels of PtRab5, Ptα2M2, and PtMBL in the PtCLec2-knockdown crabs.

### 2.13. PtCLec2 May Regulate AMP Expression via Immune Pathways

**Figure 16:**
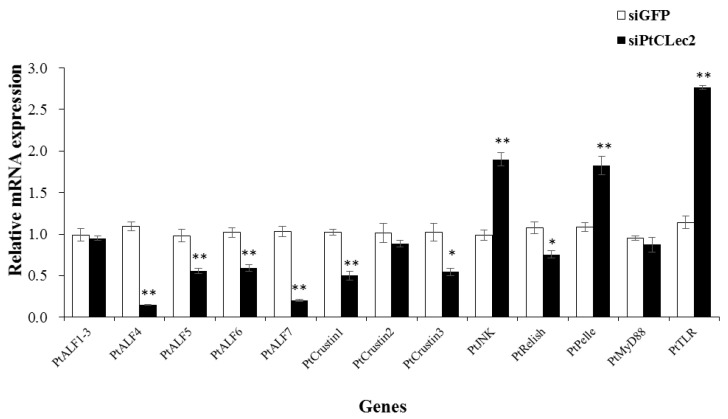
Expression levels of AMPs and genes related to immune pathways after PtCLec2 RNAi. Error bars represent the mean ± S.D. (*n* = 3). Significant differences compared with control are denoted with asterisks (* *p* < 0.05, ** *p* < 0.01).

To investigate whether PtCLec2 could regulate AMP expression, the expression of eight AMP genes (including five ALFs and three Crustins) were detected in the crabs after RNAi of PtCLec2. The relative expression levels of PtALF4-7, PtCrustin1, and PtCrustin3 were significantly inhibited in the PtCLec2-knockdown crabs (Figure 16). We further detected the expression of several genes related to the IMD, Toll, and JNK pathways, and found knockdown of PtClec2 could downregulate the expression of PtRelish in the IMD pathway. However, the expression of key genes PtJNK, PtPelle, and PtTLR involved in the JNK and Toll pathways were remarkably enhanced.

### 2.14. Effect of PtCLec2 Interference on proPO System

**Figure 17:**
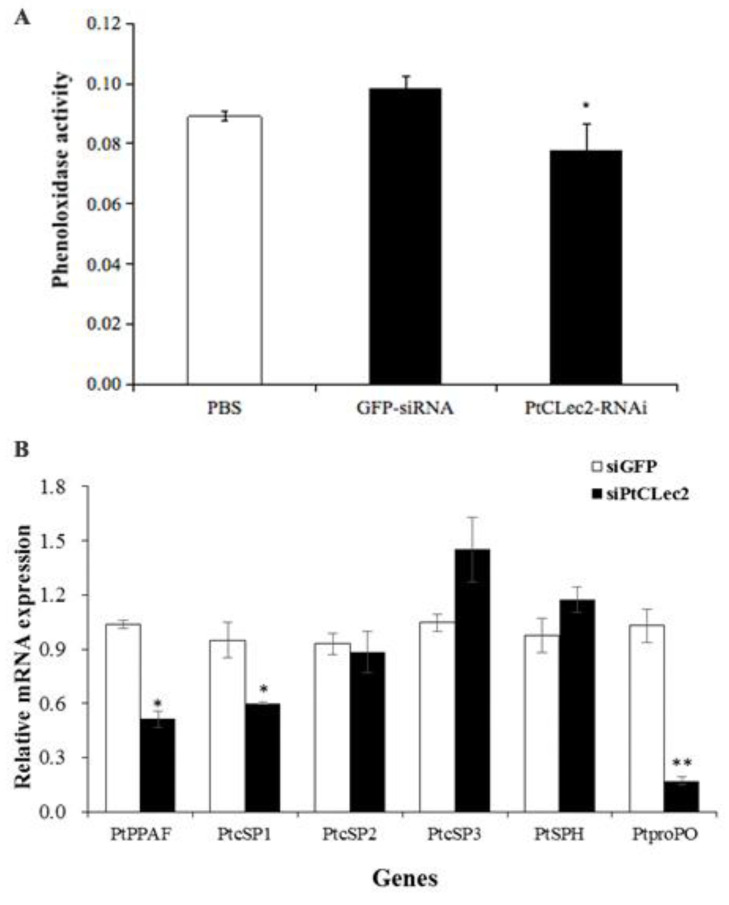
Phenoloxidase activity (**A**) and expression of proPO system genes (**B**) after PtCLec2 RNAi. Error bars show the mean ± S.D. (*n* = 3). Significant differences compared with control are denoted with asterisks (* *p* < 0.05, ** *p* < 0.01).

To investigate the possible involvement of PtCLec2 in the proPO system, the PO activity was determined in the hemolymph of PtCLec2-knockdown crabs (Figure 17A). The results show RNAi-mediated PtCLec2 knockdown could significantly reduce the total PO activity by 20.8% compared with that in the siGFP injection group. We further detected the expression levels of proPO system-related genes and found that PtPPAF, PtcSP1, and PtproPO were significantly inhibited compared with the control group, while there was no obvious change on the expression of PtcSP2, PtcSP3 and PtSPH in the PtCLec2-knockdown crabs (Figure 17B).

## 3. Discussion

In the present study, a C-type lectin named PtCLec2 was characterized in *P. trituberculatus*. The CRD of PtCLec2 was found to be the typical long form including six cysteine residues that could form three disulfide bonds. Meanwhile, PtCLec2 with the QPD motif in Ca^2+^-binding site 2 displayed high sequence similarity to other crustacean C-type lectins [22,23]. Phylogenetic analysis revealed PtCLec2 was closely related to C-type lectins of *S. paramamosain* and *E. sinensis* but clearly distinct from other *P. trituberculatus* lectins. Both the sequence similarity and clustering analysis indicate that PtCLec2 belongs to the C-type lectin family.

Hepatopancreas is the main expression tissue of most reported crustacean C-type lectins [24]. However, in our study, PtCLec2, similar to MrLec from *Macrobrachium rosenbergii* [25], was primarily expressed in intestine. The intestine has been reported to play critical roles not only in digestion but also in immune responses in many insects [26]. The highest expression of PtCLec2 in intestine suggests that intestine might participate in the crustacean immune response. The expression levels of PtCLec2 were relatively higher in gill and eyestalk, which was also reported in CgCLec-3 from *C. gigas* [27] and PcLec6 from *P. clarkii* [28]. These results indicate that PtCLec2 could take part in the immune defense of gill and eyestalk. After challenge with Gram-negative bacteria and fungi, the transcripts of PtCLec2 were increased significantly in a short time, suggesting that PtCLec2 might be recruited for the defense against invading pathogen. The similar results were also found in EsCTL [29] and EsGal from *E. sinensis* [30]. In addition, the expression of PtCLec2 was not significantly upregulated until 48 h post *M. luteus* challenge, suggesting that PtCLec2 might respond to invading *M. luteus* less sensitively than to *V. alginolyticus* and *P. pastoris*.

The key characterization of C-type lectins is recognition and non-covalent binding of PAMPs on pathogen surface, and agglutination by binding to glycoproteins and glycoconjugates on cell surface [31]. In the present study, rPtCLec2 manifested direct binding to LPS, PGN, and GLU, indicating that PtCLec2 could have a broader PAMP-binding spectrum. The binding affinities of rPtCLec2 to LPS and GLU were higher than to PGN, which agreed with the results of CfLec-4 of *Chlamys farreri* [32] and AiCTL-7 of *Argopecten irradians* [33]. However, the agglutination assay showed rPtCLec2 preferred to bind LPS rather than PGN and GLU. This inconsistency of binding and agglutinating activity of C-type lectins was also found in FcLec4 of *F. chinensis* [22] and Ha-lectin of *Helicoverpa armigera* [34]. All these results suggest that PtCLec2 is an LPS-preferred binding and agglutinating lectin.

Similar to EsLecF from *E. sinensis* [35] and FmLC3 from *F. merguiensis* [36], rPtCLec2 could bind the tested microorganisms in the presence of Ca^2+^, suggesting the broad microbial-binding spectrum of PtCLec2. The relatively weak affinity rPtCLec2 to fungi was consistent with the results of EsLecH from *E. sinensis* [37] and PmCLec from *P. monodon* [38]. As reported in Fc-Lec2 from *F. chinensis* [39] and AjCTL-1 from *Apostichopus japonicus* [40], rPtCLec2 also exhibited calcium-dependent agglutinating capability to bacteria but not to fungi. The observation that rPtCLec2 could bind but not agglutinate fungi was also found in FcLec4 from *F. chinensis* [22], further supporting the different mechanisms between binding and agglutinating activity. Moreover, rPtCLec2 could highly agglutinate rabbit erythrocytes by addition of Ca^2+^, which was in agreement with reports on AiCTL-6 from *A. irradians* [41] and PtCTL4 from *P. trituberculatus* [21]. Therefore, PtCLec2 might be a microorganism-sensing and Ca^2+^-dependent PRR and play a crucial role in immune recognition.

Classical vertebrate QPD and EPN motifs in the CRD have specificity for galactose and mannose, respectively. However, the carbohydrate-binding specificity of C-type lectins is not conserved in invertebrates [4]. In our study, rPtCLec2 with QPD motif could bind both d-galactose and d-mannose, which was similar to that reported in some invertebrates, for instance, LvCTL1 from *Litopenaeus vannamei* [5] and AiCTL-3 from *A. irradians* [42] with EPN motif, and PtCLec1 from *P. trituberculatus* [43] and FmLC6 from *F. merguiensis* [44] with QPD motif. These patterns indicate that invertebrate QPD or EPN motif of C-type lectins have more diverse carbohydrate recognition specificity. It might be important for invertebrates to rely solely on innate responses against invading pathogens.

The opsonization in hemolymph could contribute to the resistance of invading bacteria and activate the phagocytic efficiency [45]. Coincident with FmLdlr from *F. merguiensis* [12] and PcLec6 from *P. clarkii* [28], rPtCLec2 could directly promote the clearance efficiency against *V. alginolyticus* in vivo. Furthermore, rPtCLec2 could substantially increase the phagocytic activity of hemocytes, and RNAi of PtCLec2 could result in a lower expression of phagocytosis-associated genes, in accordance with reports on SpCTL-B from *S. paramamosain* [46] and PcLec3 from *P. clarkii* [13]. These results suggest that PtCLec2 could perform as an opsonin in the regulation of cell-mediated immune responses. Furthermore, rPtCLec2 could obviously inhibit the growth of Gram-negative and Gram-positive bacteria in a dose-dependent manner, indicating that PtCLec2 is a potent antibacterial protein. Recombinant proteins of EsLecF from *E. sinensis* [35] and CgCLec-4 from *C. gigas* [47] showed similar antibacterial activity.

To study the mechanism of PtCLec2 in regulation of antibacterial immunity, the expression of AMPs and several genes of immune signaling pathways were detected. In contrast to that reported in PtCLec1 [43], the expression of ALFs and Crustins were obviously suppressed after RNAi of PtCLec2, suggesting the positive regulatory roles of PtCLec2 in AMP production. Similar results were also observed in EsLecH from *E. sinensis* [37] and MnCTL from *M. nipponense* [48]. Furthermore, the changing pattern of AMP genes was in accordance with PtRelish expression, indicating PtCLec2 might regulate AMP synthesis through IMD pathway. However, the expression of PtJNK in JNK pathway, and PtPelle and PtTLR in Toll pathway genes were significantly increased in the PtCLec2-knockdown crabs. These results suggest PtCLec2 might induce multiple signaling pathways to participate in AMP regulation.

The current study showed that the PO activity detected in hemolymph and the expression of proPO system-related genes had obviously decreased in the PtCLec2-knockdown crabs, suggesting PtCLec2 could activate the proPO system. The results were similar to that found in EsLecB from *E*. *sinensis* [16] and PcLec2 from *P. clarkii* [17] but in contrast to that reported in PtCLec1, which could serve as a putative negative regulator of the proPO system [43]. The complement-like component α2M could regulate activation of the proPO system [6,49]. Our results revealed that knockdown of PtCLec2 led to a significant downregulation of complement-like component Ptα2M1 but not Ptα2M2, further supporting the regulation of PtCLec2 in activating the proPO system.

## 4. Materials and Methods

### 4.1. Gene Cloning and Bioinformatic Analysis

Partial cDNA sequence of PtCLec2 was obtained from transcriptome sequencing of *P. trituberculatus*. The complete cDNA sequence of PtCLec2 was amplified by rapid amplification of cDNA end (RACE) PCR with specific primers (Table 2). BLAST (available online: http://blast.ncbi.nlm.nih.gov/Blast.cgi (accessed on 15 January 2021)) was used to analyze the cDNA sequence and deduce the amino acid sequence of PtCLec2. Domain architecture prediction of PtCLec2 was performed by SMART (available online: http://smart.embl.de (accessed on 15 January 2021)). The potential disulfide bonds were obtained by DiANNA 1.1 web server (available online: http://clavius.bc.edu/~clotelab/DiANNA (accessed on 15 January 2021)) [50]. Molecular weight (Mw) and isoelectric point (pI) were predicted by ExPASy (available online: https://web.expasy.org/compute_pi (accessed on 15 January 2021)). Multiple protein sequence alignment and phylogenetic tree construct were conducted using DNAMAN 8.0 and MEGA 5.0 [51] software, respectively.

### 4.2. Experimental Animals, Tissue Collection, and Immune Challenge

Healthy *P. trituberculatus* (120 ± 5 g) were collected from an aquatic market in Qingdao, Shandong Province, China, and acclimated in seawater (15 ± 1 °C) for 7 days prior to the experiment. After one week of culture in the laboratory, the brain, eyestalk, gill, heart, hemocytes, hepatopancreas, intestine, muscle, stomach, testis, and thoracic ganglia were dissected from healthy crabs (*n* = 5) for tissue distribution expression analysis. For the pathogen challenge experiments, three microorganisms *V*. *alginolyticus*, *M*. *luteus*, and *P*. *pastoris* were separately prepared and resuspended in PBS at the final concentration of 3 × 10^8^ CFU mL^−^^1^. The experimental and control group crabs were injected with 100 μL microbial suspension and sterile PBS, respectively. Hemocytes of challenged crabs were respectively collected at 0, 2, 4, 8, 12, 24, 48, and 72 h post-injection. Briefly, hemolymph was extracted with an equal volume of anticoagulant solution (336 mM NaCl, 115 mM glucose, 27 mM sodium citrate, 9 mM EDTA, pH 7.0), and immediately centrifuged (800× *g*, 5 min, 4 °C) to separate the hemocytes. Total RNA from crab tissues was extracted by using TRIzol reagent (Invitrogen).

### 4.3. The Temporal mRNA Expression of PtCLec2

The mRNA expression of PtCLec2 was determined by quantitative real-time PCR (qRT-PCR) with the primers PtCLec2-qRT-F and PtCLec2-qRT-R (Table 2). The β-actin gene was used as an internal control to calibrate the cDNA template. PCR amplification was carried out in 10 μL reaction system containing 2.4 μL of sterile distilled H_2_O, 5 μL of 2× SYBR Premix Ex Taq (TaKaRa), 0.2 μL of 50× ROX Reference Dye, 0.2 μL of each primer (10 μmol L^−1^), and 2 μL of the diluted cDNA. The PCR program was 95 °C for 30 s, and 40 cycles of 95 °C for 5 s and 60 °C for 35 s. Each sample was repeated in triplicate. The relative expression level of PtCLec2 was calculated by 2^−ΔΔCt^ methods [52], and shown as mean ± S.D.

### 4.4. Expression and Purification of Recombinant PtCLec2

The sequence encoding the mature peptide of PtCLec2 was obtained using the specific primers RE-PtCLec2-F and RE-PtCLec2-R (Table 2). The purified PCR fragment was digested with restriction enzymes *Bam*H I and *Xho* I (NEB, Ipswich, MA, USA) and then ligated into pET-32a (Novagen, Germany) using T4 DNA ligase (TaKaRa). The recombinant plasmid pET-32a-PtCLec2 was verified for nucleotide sequence and subsequently transformed into *E. coli* BL21 (DE3). The empty plasmid pET-32a expressing recombinant thioredoxin (rTrx) was used as negative control. After sequencing to ensure insertion, both positive transformants of PtCLec2 and negative control were grown in 250 mL LB liquid medium with 100 μg mL^−1^ ampicillin (220 rpm, 37 °C) until OD_600_ of 0.5–0.7. IPTG was then added at a final concentration of 1 mmol L^−1^ and the culture was grown at 37 °C with agitation at 220 rpm for an additional 6 h.

Cells were obtained by centrifugation at 8000× *g* for 5 min at 4 °C, and resuspended in buffer I (300 mM NaCl, 50 mM sodium phosphate, pH 7.0), and sonicated for 30 min at 4 °C. Cell lysate was centrifuged at 8000× *g* (4 °C, 15 min) to collect the inclusion bodies, which were then dissolved in buffer II (300 mM NaCl, 50 mM sodium phosphate, 8 M urea, pH 7.0). TALON metal affinity resin (Clontech) was used to purify the recombinant PtCLec2 (rPtCLec2) and rTrx proteins. The purified proteins were refolded in gradient urea-TSB glycerol buffer according to our previous study [43]. The purified proteins were detected by 15% SDS-PAGE and visualized with Coomassie brilliant blue R250. The concentration of rPtCLec2 and rTrx were measured by BCA Protein Assay Kit (Beyotime).

### 4.5. PAMP-Binding Assay

ELISA was used to examine the binding activity of rPtCLec2 to PAMPs according to a previous method [43]. In brief, microtiter plates were coated overnight at 4 °C with 100 μL of lipopolysaccharide, peptidoglycan, and glucan (Sigma-Aldrich, St. Louis, MO, USA) (20 μg). The wells were washed twice with PBST and blocked by 3% BSA in PBS for 1 h at 37 °C. Then 100 μL of rPtCLec2, diluting with PBS within 0.1 mg mL^−1^ BSA and 10 mmol L^−1^ CaCl_2_ were added to the wells and incubated for 3 h at room temperature. The serial concentrations of rTrx served as control. After washing of unbound proteins, the wells were incubated with 100 μL of mouse anti-His tag antibody (1:1000 diluted, TransGen, Beijing, China) as the primary antibody for 1 h at 37 °C for. The plates were washed and incubated with 100 μL of goat-anti-mouse Ig-HRP conjugate as the second antibody (1:2000 diluted, TransGen, China) for 1 h at 37 °C. After the last wash, the color reaction was detected using TMB substrate kit (Sangon, Shanghai, China) and the absorbance was measured at 450 nm. Each experiment was repeated in three times. The results of ELISA index (EI) were recorded as OD_sample_/cut off, where the cut off was mean OD of negative controls + three S.D.s at every point. Samples with EI > 1.0 were determined to be positive.

### 4.6. Microbial-Binding Assay

Five microorganisms, including Gram-negative bacteria *P*. *aeruginosa* and *V. alginolyticus*, Gram-positive bacteria *S*. *aureus* and *M. luteus*, and fungus *P. pastoris*, were used to determine the binding activity of rPtCLec2 according to the previous methods [53]. After cultivation to logarithmic growth phase, both bacterial and fungus were collected by centrifugation at 10,000 rpm for 1 min. The microbial pellets were fixed with 37% formaldehyde for 1 h and washed twice with PBS. Cell suspension was incubated with rPtCLec2 with the addition of CaCl_2_ (10 mmol L^−1^) for 30 min with gentle rotation. rTrx incubated microorganisms were employed as negative control. The supernatant was removed after centrifugation. After washed twice by PBS, the bound proteins were eluted from microorganism by SDS-PAGE loading buffer. The eluted, supernatant, and wash fractions were analyzed by 15% SDS-PAGE.

### 4.7. Microbial Agglutination and Inhibition Assay

Five aforementioned microorganisms were used to test the agglutination ability of rPtCLec2 according to a previous study [30] with minor modifications. Microorganisms were grown to mid-logarithmic phase in the corresponding growth medium and collected by centrifugation at 10,000 rpm for 1 min. The microorganisms were washed with PBS, resuspended to 10^7^ cells mL^−1^, and stained with FITC. The FITC-labeled microorganisms were washed with PBS until the supernatant is clear of obvious fluorescent material. A volume of 10 μL microbial suspension was mixed with 25 μL rPtCLec2 with the addition of CaCl_2_ (10 mmol L^−1^) or EDTA (10 mmol L^−1^). After rotation in the dark for 2 h, the mixture (5 μL) was observed under fluorescence microscopy. rTrx and PBS were served as a negative and blank control, respectively.

Seven carbohydrates were used to measure the carbohydrate-binding specificity of rPtCLec2, including d-galactose, d-glucose, d-mannose, sucrose, LPS, peptidoglycan, and glucan (Sigma-Aldrich, USA). rPtCLec2 (25 μL, 100 μg/mL) was premixed with 20 μL of various carbohydrates ranging from 25 to 200 mmol L^−1^ (2-fold diluted) for 30 min with the addition of 10 mmol L^−1^ CaCl_2_. Then, FITC-labeled *P. aeruginosa* suspension was added to the mixture for 1 h. The inhibitory activity was displayed as the minimum concentration of carbohydrate that could completely inhibit bacterial agglutinating activity.

### 4.8. Hemagglutination Assay

The hemagglutination activity of rPtCLec2 was determined as previously described [54]. Briefly, rabbit erythrocytes (Yuanye, Shanghai, China) were resuspended in 2% TBS-Ca buffer (50 mM Tris-HCl, 10 mM CaCl_2_, 100 mM NaCl, pH 7.5). Two-fold serially diluted rPtCLec2 (30 μL) mixed with erythrocyte suspension (30 μL) were added in a V-type microtiter plate. rTrx dissolved in TBS-Ca buffer was served as negative control. Erythrocyte suspension with rPtCLec2 containing EDTA (10 mM) was used to determine whether hemagglutination was Ca^2+^-dependent. After incubation for 45 min at 37 °C, the hemagglutination extent was checked by light microscope.

### 4.9. Antimicrobial Activity

Antimicrobial activity of rPtCLec2 was tested against five mentioned microorganisms using a liquid phase assay with minor modifications [55]. Microorganisms in mid-logarithmic phase were centrifuged and resuspended in Tris-HCl buffer at 1 × 10^3^ CFU mL^−1^. A total of 50 μL of serially two-fold diluted rPtCLec2 in Tris-HCl buffer and 50 μL of microorganism suspension were added to the 96-well plate and incubated at room temperature for 2 h. rTrx and Tris-HCl were used as negative and blank control, respectively. Then, 150 μL corresponding medium were added into the cell suspension and the mixtures were incubated overnight at the corresponding temperatures. Absorbance was determined at 560 nm for yeast or 600 nm for bacteria by microplate reader. Each measurement was recorded in triplicate.

### 4.10. In Vivo Bacterial Clearance Assay

The bacterial clearance assay was carried out according to the previously reported method [56]. *V. alginolyticus* (1 × 10^3^ CFU mL^−1^) was pre-incubated with rPtCLec2 for 1 h at 4 °C, and 100 μL of mixture was then injected into healthy crab (*n* = 3). The negative and blank control group were crabs that receiving the injection of *V. alginolyticus* pre-incubated with rTrx or PBS. The hemolymph was harvested at the time point of 15, 30, and 90 min post-injection, and immediately diluted with an equal volume of PBS. The diluted hemolymph (100 μL) was plated onto solid LB medium and incubated overnight at 30 °C. The number of bacterial colonies on the plate was counted. The assay was conducted in triplicate.

### 4.11. Phagocytosis Assay

The phagocytosis activity of rPtCLec2 was observed by microscopy and measured by flow cytometry [43]. Heat-killed *V. alginolyticus* was labeled by FITC-NaHCO_3_ solution (containing 0.1 mg mL^−1^ FITC) for 2 h. The hemocytes of crab were resuspended in anticoagulant solution and incubated with rPtCLec2 containing CaCl_2_ (10 mM) for 30 min at 18 °C. After washing with PBS, the FITC-labeled *V. alginolyticus* were incubated with the mixture of hemocytes and rPtCLec2 at 18 °C for 1 h. Then, the hemocytes were washed and resuspended with anticoagulant. A portion of hemocytes were analyzed by flow cytometry (FACSAria, BD biosciences, USA) and a total of 10,000 events were collected to evaluate the phagocytosis rate (PR). PR was defined as (phagocytic hemocytes)/(total tested hemocytes) × 100%. The other hemocytes were fixed in 4% paraformaldehyde for 30 min and observed under laser scanning confocal microscopy. The hemocytes incubated with rTrx served as negative control and each assay was repeated in triplicate.

### 4.12. RNA Interference Assay

The PtClec2-siRNA specifically targeted to PtCLec2 (Table 2) was synthesized by in vitro transcription T7 kit (Thermo, Waltham, MA, USA). The siGFP was synthesized by GFP-siRNA (Table 2) as negative control. The reaction was carried out in a system of 20 μL, containing 4 μL of 5× transcription buffer, 2 μL of TranscriptAid enzyme, 2 μL of the four NTPs, 3 μL of template, and 3 μL of RNase-free water. After incubation for 8 h at 37 °C, the transcription product was incubated for 10 min at 75 °C to end the transcription reaction. Then, DNase I and RNase T1 were added and incubated for 30 min at 37 °C. The siRNA for PtCLec2 (200 μg, 2 μg/μL) was injected into the arthrodial membrane of the last walking leg of each crab. A second injection was performed at 24 h after the first injection to enhance the interference effect of PtCLec2. The hemocytes and intestine of five crabs were individually sampled at 24 and 48 h post the second injection. Total RNA was extracted in all the groups and the knockdown efficiency of PtCLec2 gene was evaluate by qRT-PCR.

### 4.13. Expression of Immune-Related Genes in the PtCLec2-Knockdown Crabs

The expression patterns of immune-related genes were detected in the PtCLec2-knockdown crabs by qRT-PCR, including phagocytosis-related genes PtArp (actin-related protein), PtRab5 (small GTP-binding protein), and PtMyosin; proPO system-related genes PtPPAF (proPO-activating factor), PtcSP1-3 (clip domain serine protease), PtSPH (serine protease homolog), and PtproPO; complement-like genes PtTEP (thioester-containing protein), Ptα2M1 and Ptα2M2 (alpha-2-macroglobulin), and PtMBL (mannose-binding lectin); antimicrobial peptides PtALF1-7 (anti-lipopolysaccharide factor) and PtCrustin1-3; and key genes related to the IMD, Toll, and JNK pathways PtRelish, PtMyD88, PtPelle, PtTLR, and PtJNK. The primer sequences of the mentioned genes are shown in Table 2.

### 4.14. Phenoloxidase (PO) Activity in the PtCLec2-Knockdown Crabs

Hemolymph was collected from three PtCLec2-knockdown crabs without using anticoagulant solution. Briefly, hemolymph protein (2 mg) in 435 μL of Tris-HCl (10 mM, pH 8.0) was incubated with 65 μL of L-3,4-dihydroxyphenylalanine (L-dopa) (3 mg mL^−1^, Sigma-Aldrich) for 30 min at room temperature. Subsequently, 500 μL of 10% acetic acid was added to each well and the PO activity was measured by absorbance at 490 nm. The siGFP- and PBS-treated crabs were used as negative and blank control, respectively. The PO assay was conducted in triplicate.

### 4.15. Statistical Analysis

Experimental data are presented as mean ± S.D. and were subjected to one-way ANOVA using SPSS 16.0. *p* < 0.05 was considered statistically significant and *p* < 0.01 was regarded as extremely significant compared with the control group. All experiments were conducted in triplicate.

## 5. Conclusions

In the present study, a new C-type lectin with high expression in the intestine was identified from *P. trituberculatus*. PtCLec2 transcripts were obviously upregulated upon bacteria and fungi challenge. The recombinant PtCLec2 showed significant binding and agglutination abilities, and even antibacterial activities and could trigger the clearance and phagocytosis of *V. alginolyticus*. PtCLec2 has the typical QPD motif that can bind both galactose and mannose rather than galactose. In addition, PtCLec2 could regulate the expression levels of phagocytosis genes, complement-like genes, AMPs, and proPO system-related genes. Hence, PtCLec2 might not only serve as a PRR in the immune recognition but also possess potential immunoregulatory properties though its antibacterial and opsonic activities, and through activation of the proPO system.

## Figures and Tables

**Table 1:** Antimicrobial activity of rPtCLec2.

Microorganisms	MIC (μM)
Gram-negative bacteria	
*Vibrio alginolyticus*	0.86–1.74
*Pseudomonas aeruginosa*	1.74–3.50
Gram-positive bacteria	
*Micrococcus luteus*	0.43–0.86
*Staphylococcus aureus*	0.86–1.74
Fungus	
*Pichia pastoris*	Na

Na: no antimicrobial activity.

**Table 2:** Primers used in this study.

Primer Name	Forward Primer (5′–3′)	Reverse Primer (5′–3′)
cDNA cloning		
PtCLec2	CCGAGAATGCGAATCTACTTGC	TGTTTACTGTGAGCAGCAGCCAC
RACE		
PtCLec2-3P1	CGACCAACGCATCGCCACCAG	
PtCLec2-3P2	CGACATTGCCTGCTACCACAAGAAG	
PtCLec2-5P1	AGCCTGGTGGCGATGCGTTGG	
PtCLec2-5P2	TCAGCCAGTCAACCTTGCTACCTTCC	
UPM (long)	CTAATACGACTCACTATAGGGCAAGCAGTGGTATCAACGCAGAGT	
UPM (short) NUP	CTAATACGACTCACTATAGGGC AAGCAGTGGTATCAACGCAGAGT	
Recombinant expression		
RE-PtCLec2	CGCGGATCCCAAGGCCGTGTGTTGGCG	CCGCTCGAGACCAAAATGTGCTCGTGCGCG
qRT-PCR		
PtCLec2-qRT	ACAGGACAACTGGAAGGTAGCAAG	GAGAAGAACCATCCCTTGACACTG
PtcSP1-qRT	ACTATGTCCAGCCAGCGTGT	GGAAGGACTCGCGCTCATAG
PtcSP2-qRT	TAAGGACATCGGACAGGAGACACT	TAAGGAAGTGAACGCTATCTCT
PtcSP3-qRT	AAGCCAGTCGAAATACAGGAG	CAGCATCTCCTTCCCAATTCC
PtSPH-qRT	CATCCTTGACCAGCCAGCA	CCCACCCAGACACAACACA
PtMyosin-qRT	CGTTGGCGAAGTAGGAGAGT	GAACAAGAGGCGTAATGAGGT
PtRab5-qRT	AACCCAGCATCCAGTCACCC	TACCCCTAAGCCCCTCAACC
PtArp-qRT	GCTCCACCATCACCACTCTG	TTAGCCATCTCCTTCCCTGC
PtproPO-qRT	CCTCTTCTTCACGACACTCAACTG	TCACGAGATAACACAAAACGCC
PtPPAF-qRT	GGACAGGACCAAGACCCAGT	GATTTGAGAAGGAACAAGCGTG
Ptα2M1-qRT	TGTGCCTCCTACCGCCTTCC	GGTGTCCCTCTCTCTCAACTCATTT
Ptα2M2-qRT	GTGGTTGGCTACGGGACGGGT	ACGGCAATGTCATCACTGGGGAT
PtTEP-qRT	CTCTTCTCGCTGCTTTCCTTCATC	TTTTTGGGACTTTGCCACCACTT
PtCrustin1-qRT	GGCAGTTGTGGCTACCATTGT	CGCTCGGTGTAAGGTGGATAG
PtCrustin2-qRT	TGGTGATGGCGGTGGCTATG	TCCTTGGCGAACATCCTCT
PtCrustin3-qRT	AGTATCTCAGAATCGACCA	CCCTCTTAGTTTCTCTTGTT
PtALF1-3-qRT	ACGACGAGGAGGAGAAAGAGG	GGCACTGATGGTGGAAACTGA
PtALF4-qRT	GACGCTCTGAAGGACTTTATG	CGCCGAAACGCTTAGAAATAC
PtALF5-qRT	TAGTCGTGGTGAGAGGGCAA	CTTTGCTCTCTCATCAGGAC
PtALF6-qRT	CATACTCCCGTGAACTCCTA	CAGACCGTGATTTGTGGAG
PtALF7-qRT	GCATTTTCTATTTTCCTATCC	GCATGAGTCTTGTATATTTGG
PtMBL-qRT	GGCACCGTCGGTCATCCAAC	GCTGGCTGCGACCAAACCTT
PtJNK-qRT	AGTGTGGCGGCTCAGCTGTT	CTCCACTCCGACTGCCTCGC
PtRelish-qRT	CCAGAGTACGCAAGCCACATCAC	CCGCAGCACCACCTTGTTCAG
PtPelle-qRT	ACTCTTGCCTTCCCTTGCTAAC	ACTGACCATGAATCATACCCCTG
PtMyD88-qRT	GGTCCTTGAAGCAACAGGTGGTAG	AGTGCTGGCTGACTAGGAGATGAC
PtTLR-qRT	CATTGAGGACAGCCACAGGAC	TGGTAGAGAGGTACAGCTTGAGTTC
β-actin-qRT	TCACACACTGTCCCCATCTACG	ACCACGCTCGGTCAGGATTTTC
RNAi		
PtCLec2-siRNA	GACGCAACAUCACGUACAU	
GFP-siRNA	GGAGGACGGCAACAUCCUG	
Sequencing		
M13	CGCCAGGGTTTTCCCAGTCACGAC	GAGCGGATAACAATTTCACACAGG
T7	TAATACGACTCACTATAGGG	GCTAGTTATTGCTCAGCGGT

The *Bam*H I and *Xho* I sites are underlined.

## Data Availability

The data presented in this study are available on request from the corresponding author.
